# Chronic Allograft Nephropathy—A Narrative Review of Its Pathogenesis, Diagnosis, and Evolving Management Strategies

**DOI:** 10.3390/biomedicines13040929

**Published:** 2025-04-09

**Authors:** Matthew Pittappilly, Mohammed Sharshir, Anil Paramesh

**Affiliations:** 1 Department of Nephrology, Tulane Transplant Institute, East Jefferson Hospital, Tulane University School of Medicine, New Orleans, LA 70112, USA; mpittappilly@tulane.edu (M.P.); msharshi@tulane.edu (M.S.); 2 Department of Surgery, Tulane Transplant Institute, East Jefferson Hospital, Tulane University School of Medicine, New Orleans, LA 70112, USA

**Keywords:** chronic allograft nephropathy, chronic kidney disease, kidney allograft failure, acute rejection, chronic rejection, interstitial fibrosis and tubular atrophy

## Abstract

Chronic allograft nephropathy is the leading cause of kidney allograft failure. Clinically, it is characterized by a progressive decline in kidney function, often in combination with proteinuria and hypertension. Histologically, interstitial fibrosis and tubular atrophy, along with features of glomerulosclerosis with occasional double contour appearance, arteriolar hyalinosis, and arteriosclerosis, are characteristic findings. The pathophysiology, though complex and incompletely understood, is thought to involve a sequence of immunologic and non-immunologic injuries eventually leading to tissue remodeling and scarring within the graft. The optimal strategy to prevent chronic allograft nephropathy is to minimize both immune- and non-immune-mediated graft injury.

## 1. Introduction

Kidney transplantation is the treatment of choice for end-stage kidney disease (ESKD) offering cost, quality of life, and survival benefits as compared to maintenance dialysis. Over the past few decades, short-term graft survival has improved, which has been mainly attributed to the prevention of acute rejection with the introduction of new immunosuppressive regimens and targeted therapies. Although the rate of early allograft loss has reduced over this time, the longevity of the graft has not changed as much. While acute rejection plays a significant role in short-term graft survival, multiple factors contribute to late graft failure.

Chronic allograft nephropathy (CAN), also referred to as interstitial fibrosis and tubular atrophy (IFTA), is an incompletely understood histopathologic diagnosis that is a major cause chronic allograft dysfunction and late graft loss in kidney transplant patients. In the era of more effective immunosuppression and improved rates of early graft survival, CAN has become the most prevalent cause of renal allograft loss after the first year. Several factors contribute to CAN and its histologic occurrence is thought to represent an accumulative burden of pathologic injuries leading to a gradual and sustained loss of kidney function.

## 2. Risk Factors

Immunologic as well as non-immunogenic factors are presumed to contribute to CAN [1].

### 2.1. Immunologic Factors

#### 2.1.1. Acute Rejection

Acute rejection is a significant risk factor associated with the development of chronic allograft nephropathy and late graft loss. Several factors, including the type, severity, timing, and persistence of inflammation, significantly influence the progression of CAN. In particular, the type, severity, timing, and persistence of acute rejection post-transplant all play an important role in the development of CAN. Acute vascular rejection and severe acute cellular rejection, defined by the need for anti-lymphocyte therapy, have been shown to be associated with the onset of histologic CAN [2]. Additionally, late acute rejection, occurring more than 3–4 months after transplantation, as well as the persistence of graft inflammation, have been strongly associated with the development of CAN [3].

#### 2.1.2. Subclinical Rejection

Subclinical rejection (SCR) is also known to be associated with CAN. In a long-term study evaluating the progression of SCR, Nankivell et al. [2] showed that SCR was common early post-transplantation. The persistence of graft inflammation on subsequent biopsy specimens was associated with a lower GFR and contributed to CAN. Many other studies have also demonstrated this association [3,4,5].

#### 2.1.3. Chronic Rejection

Failure to resolve acute inflammation can predispose to CAN. Repeated episodes of acute rejection result in sustained immunologic activity against the allograft through both cellular and humoral mechanisms, including the development of donor-specific antibodies (DSAs). Over time, this leads to progressive damage and the loss of kidney function.

#### 2.1.4. HLA Mismatches

With modern immunosuppression, both well- and poorly HLA-matched kidneys have similar short- and medium-term allograft survival rates. However, HLA mismatching remains an important factor that impacts long-term allograft survival [6,7].

#### 2.1.5. Immunosuppressive Regimens

Adequate immunosuppression is necessary to prevent both subclinical and acute rejection. While immunosuppressive drugs, such as calcineurin inhibitors (e.g., cyclosporine and tacrolimus), are essential for preventing rejection, their prolonged use is associated with nephrotoxicity. Newer immunosuppressive agents, like mycophenolate mofetil, are associated with a reduction in the incidence of both early and late rejection [8,9], whereas use of cyclosporine is associated with a higher rejection risk.

### 2.2. Non-Immunologic Factors

Both donor and recipient non-immunologic factors can promote chronic injury and contribute to CAN. These include the following [10]:

- Arterionephrosclerosis;
- Prolonged cold ischemia time;
- Non-living donation;
- Increasing donor age;
- Donor–recipient size discrepancy;
- Nephrocalcinosis related to preexisting hyperparathyroidism;
- Calcineurin inhibitor nephrotoxicity;
- Recurrent glomerulonephritis;
- Infections (BK virus, cytomegalovirus, and UTIs).

## 3. Pathogenesis

Though the precise mechanism of CAN is not completely understood, its occurrence is thought to represent an accumulation of immune and non-immunologic challenges to the kidney along with the kidney’s repair mechanisms.

**Figure 1:**
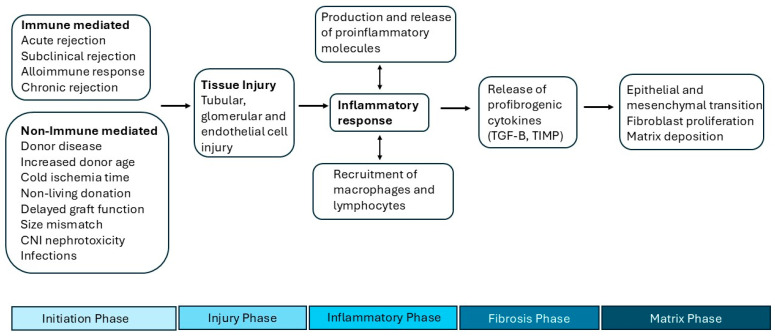
Schema of pathogenesis of CAN. TGF-B, transforming growth factor-beta; TIMP, tissue inhibitor of metalloproteinases.

The initial insult occurs even before the alloimmune response begins. Donor-related alterations in situ, including intrinsic changes seen in older-age kidneys, previous hypertension of the donor, brain death, and donor vascular disease, reflect early changes that predispose to CAN independently of other potential risk factors [11]. During procurement and implantation, the ischemia–reperfusion injury causes an increased alloimmune response in the graft following revascularization [12]. During the first few weeks following transplant, the series of insults continue. Acute tubular injury with resulting delayed graft function, acute rejection, subclinical rejection, infections, recurrence of native kidney disease, obstruction, and CNI nephrotoxicity can occur, further adding to the allograft injury. This all occurs in the setting of a pro-inflammatory environment in the transplanted kidney. Furthermore, in the case of older donors who are faced with reduced renal reserve, the capacity for repair is limited, leading to reduced kidney function [13]. The proposed general pathophysiology of CAN is demonstrated in Figure 1.

The series of renal injuries results in tissue injury and an inflammatory response. Neutrophils are typically the first cells recruited. They release pro-inflammatory mediators like ROS and proteolytic enzymes and produce various cytokines and chemokines, further attracting other immune cells to the site of inflammation. Macrophages and T and B lymphocytes follow, infiltrating injured tissue and secreting fibrogenic cytokines such as TGF-B and tissue inhibitor of metalloproteinases (TIMP) [14]. The inflammatory cascade results in the downstream activation and proliferation of mesenchymal cells such as myofibroblasts and smooth muscle cells in the vascular wall that promote the fibrogenic milieu [15]. Excessive collagen production and matrix deposition cause a reduction in peritubular blood flow and damage to the peritubular capillaries, resulting in capillary rarefaction [16]. This ultimately leads to tubular hypoxia and a loss of nephrons, resulting in interstitial fibrosis and tubular atrophy, the non-specific pattern of injury typical of CAN.

It should be noted that these events are dynamic and often occur simultaneously. It is still under debate whether the initial allograft injury takes place in the tubular cells or the vascular wall. However, findings suggest that in the early post-transplant period, damage to the tubulointerstitial cells dominates. This often occurs in the setting of the ischemia–reperfusion injury, acute tubular necrosis, acute rejection, and CNI nephrotoxicity. Late glomerular and microvascular injury then takes precedence, related to CNI nephrotoxicity, chronic rejection, hypertension, and recurrent glomerulonephritis [17].

A recurring pattern of non-specific injury can trigger inflammation, heighten allorecognition, and cause further damage, potentially creating a self-sustaining cycle that drives continuous damage. In addition, because the changes linked to chronic allograft nephropathy also occur in aging kidneys with limited cell cycle capacity, the cumulative burden of injury exhausts the ability of key cells to repair and remodel, otherwise known as renal senescence [18]. This is believed to contribute to the histopathological lesions characteristic of CAN.

## 4. Histology

**Figure 2:**
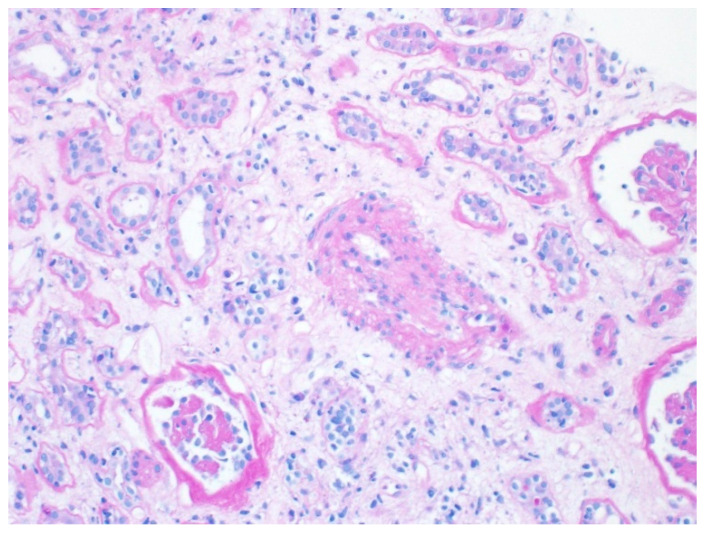
Histologic manifestation of CAN. Periodic acid Schiff stain, ×200. Image obtained with permission from Arkana Laboratories.

The characteristic features that define CAN include interstitial fibrosis and tubular atrophy (IFTA). The pathologic changes may also have features of glomerulosclerosis with occasional double contour appearance, arteriolar hyalinosis, and arteriosclerosis [17] (Figure 2).

The severity of CAN be graded quantitatively based on the severity of IFTA and the percentage of cortical parenchyma involved as detailed in Table 1 [19].

Studies involving protocol biopsies of renal allografts show that histopathologic changes consistent with CAN, although often mild, can be present in as much as 30–40% of grafts at the time of transplantation [20]. These findings can be seen even in recipients of living donor allografts [21].

New histopathologic lesions begin to develop around 3 months following transplantation and gradually progress over time [22,23]. In a pivotal study looking at the natural history of CAN, Nankivell et al. [2] showed that the progression of histologic changes associated with CAN could be divided into two distinct phases. An initial phase was observed in the first year post-transplant, marked by the onset of tubulointerstitial damage. In this phase, the damage resulted primarily from immunologic factors, including acute and subclinical rejection. Beyond one year, the histologic patterns of injury changed, now marked by arteriolar hyalinosis with vessel narrowing, glomerulosclerosis, and additional tubulointerstitial damage. In this phase, acute inflammatory activity was generally low or non-existent and true chronic rejection occurred infrequently. The damage was thought to be related to calcineurin-inhibitor nephrotoxicity, which became more common by ten years.

However, in a subsequent study looking at the histologic changes following kidney transplantation, Stegall et al. [24] demonstrated that most renal allografts exhibit only mild histologic damage at one and five years following transplantation. Their findings suggest that it is uncommon for severe histologic changes to be present in the first five years after transplantation. The discrepancy in findings seen between the two studies likely reflect the differences in study population, immunosuppression used, and post-transplant complications.

## 5. Diagnostic Evaluation

Chronic allograft nephropathy should be considered in kidney transplant recipients with a slow but progressive reduction in allograft function often accompanied by hypertension and increasing proteinuria, typically in the non-nephrotic range.

The goal of diagnosis should be to identify the potential causes of progressive kidney dysfunction. The following testing represents one such approach:

- A kidney ultrasound with Dopplers should be performed to assess blood flow and echogenicity of the allograft. A resistive index of 80 or higher has been identified as a strong predictor of long-term allograft failure [25]. While an ultrasound can offer valuable diagnostic information for allografts with chronic dysfunction, it is not a reliable screening test for chronic allograft dysfunction. Acute vascular rejection with endarteritis and chronic allograft nephropathy can both present with elevated resistive index and only a renal biopsy can distinguish them.
- Proteinuria should be assessed by a spot urine protein-to-creatinine ratio. If proteinuria > 1 gm/day is confirmed, a kidney biopsy should be performed.
- The presence of donor-specific antibodies (DSAs) should be assessed. The development of DSAs post-transplant is associated with poor outcomes in kidney transplantation [26,27,28]. Traditionally, DSAs have been associated with antibody-mediated rejection (ABMR) [29]. However, several studies have indicated that the development of DSAs signals a more complex immune response, reflecting the involvement of both humoral and cellular immunity [30,31].
- BK polyoma virus (BKPyV) should be assessed by measuring the BKPyV viral load. The reported incidence of BK nephropathy ranges from 1–10% in the current era of more effective immunosuppression [32,33]. BK nephropathy can result in severe damage to the allograft, potentially causing graft failure [34]. Histologically, it typically presents as a mononuclear cell interstitial infiltration with tubulitis. If left untreated, it can progress to a histologic pattern of tubular atrophy and chronic fibrosis [35].
- Although practices may vary between transplant centers, a kidney biopsy is recommended to confirm the diagnosis, exclude other possible conditions, and provide prognostic insights to aid in patient counseling.

Though an increase in serum creatinine is often the first sign alerting clinicians to CAN, graft failure is typically unavoidable once creatinine levels begin to rise [36]. Research has shown that serum creatinine tends to underestimate the decline in glomerular filtration rate (GFR) [37]. More advanced techniques for identifying reversible causes of CAN at an earlier stage may enable timely intervention, potentially slowing disease progression. These approaches include those in the following sub-sections.

### 5.1. Protocol Biopsies

In recent years, protocol biopsies have gained traction as a strategy for the early detection of acute or chronic graft dysfunction, aiming to improve long-term graft survival. Research has shown that these biopsies can identify subclinical rejection (SCR) at an early stage, a histologically defined acute rejection occurring without a rise in serum creatinine or proteinuria [38,39,40]. The incidence of SCR is difficult to elucidate due to variations in immunosuppressive regimens across transplant centers and differences in recipient immunologic risk factors. The initial protocol biopsies conducted by Rush et al. [41] reported a 30% incidence of SCR. Subsequent studies have shown a variable incidence of SCR from 2.6% to 25% within the first year [8]. Evidence suggests that if left untreated, SCR can contribute to chronic histologic changes and declining renal function. A longitudinal study by Nankivell examining the natural course of untreated SCR revealed that 45.7% of biopsy specimens showed SCR at three months, which was associated with increased interstitial fibrosis and tubular atrophy at 12 months [2]. Several studies suggest that early recognition and treatment of SCR has been shown to improve long-term renal outcomes [4,42,43]. Despite this, few studies have examined the long-term impact of SCR beyond the first year post-transplant. In an observational study, Loupy et al. [44] evaluated the long-term impact of early SCR detection on kidney allograft survival in a cohort of 1307 transplant recipients. Their findings showed that at eight years post-transplant, patients with subclinical antibody-mediated rejection (SC-AMR) had the lowest graft survival (56%) compared to those with subclinical T-cell-mediated rejection (SC-TCMR) (88%) and those without rejection (90%). These results suggest that SC-TCMR and SC-AMR have distinct effects on long-term allograft survival. Another longitudinal study by Mehta et al. [45] examined the impact of SC-TCMR identified in early protocol biopsies on future immunologic events and graft survival. Their findings indicated a twofold increased risk of graft loss and a fourfold higher risk of subsequent clinical rejection. The differences in methodology between these studies may explain their contrasting results. However, both studies underscore the necessity of identifying patients with SCR using protocol biopsies to develop targeted strategies for preventing alloimmune injury and improving long-term graft survival.

### 5.2. Urinary Biomarkers

Specific urinary proteins have been examined by several research groups as noninvasive biomarkers for detecting acute rejection [46]. Urinary chemokines such as CCL2, CXCL9, and CXCL10 act as key signaling molecules that recruit immune cells to the transplanted kidney and have been identified as biomarkers of acute rejection [47]. In Clinical Trials in Organ Transplantation-04 (CTOT-04), Sunthanthiran et al. [48] investigated whether urinary-cell levels of messenger RNA (mRNA) ascertained at the time of biopsy from 485 kidney transplant recipients correlated with allograft rejection. A three-gene diagnostic signature was developed using CD3*ε*, CXCL10, and 18S ribosomal RNA which was able to discriminate between biopsy specimens showing acute cellular rejection and those showing no rejection with an AUROC of 0.85 (*p* < 0.001). It also predicted future episodes of acute cellular rejection as early as 80 days before they developed. Clinical trials looking at urine CXCL10 chemokine monitoring post-renal transplant are ongoing [49].

### 5.3. Novel Tissue Diagnostics

Measuring mRNA transcripts in biopsy tissue has been shown to diagnose and monitor rejection by identifying specific gene expression patterns associated with different rejection phenotypes occurring in the transplanted kidney, particularly in detecting antibody-mediated rejection (ABMR) where traditional biopsy methods might not be conclusive. The Molecular Microscope Diagnostic (MMDx) system is one such approach that uses gene expression by microarrays to measure transcript changes in biopsies compared to a reference set using machine learning-derived algorithms. It has been validated in multicentric prospective trials to identify both ABMR and TCMR [50,51]. The lack of widespread adoption of the MMDx system is likely a result of costs and reported discrepancy with standard histologic diagnosis [52]. More thorough validation is necessary to confirm its advantage over histology [53].

Gene expression profiles offer valuable insight into the molecular processes underlying immune injury before any detectable damage occurs. The Chronic Allograft Damage Index (CADI) score is a histologic measure of the level of chronic damage in the transplanted kidney. The Genomics of Chronic Allograft Rejection (GoCAR) study utilized microarrays to identify genes associated with the CADI score at 12 months, based on tissue samples obtained three months after transplantation [54]. A set of 13 genes were identified that was independently predictive for the development of chronic damage at 1 year. Using the blood samples from the GoCAR study as a training set, a recent multicenter validation study [55] identified a 17-gene set, commercially available as Tutivia, that outperformed serum creatinine to discriminate rejection. Further study is needed to determine its clinical implications.

### 5.4. IF by Morphometry

The precise assessment of IFTA on histology pathology still proves challenging. There is high inter- and intra-observer variability in scoring for IF and TA in current clinical practice. Studies have shown that IFTA foci density by morphometric testing is a highly prognostic marker of progressive CKD and ESKD [56,57]. In a recent study, Denic et al. assessed the use of IFTA foci density in kidney transplant recipients transplanted between 2000 and 2013 who had a 5-year surveillance kidney biopsy and subsequent follow-up [58]. Compared with the current Banff classification for grading kidney fibrosis, the morphometric characterization of IFTA foci density more strongly predicted allograft failure. Morphometry may be too time consuming for routine biopsies so further study is needed to evaluate its clinical implications to identify at-risk patients.

### 5.5. Blood Biomarkers

Multiple blood biomarkers have been evaluated to assess for renal allograft injury following transplant. Donor-derived cell-free DNA (ddcfDNA) has been found to be increased in acute rejection. Results from the DART trial [59] validate that at a 1% diagnostic cut off, ddcfDNA can discriminate active rejection from no rejection but the clinical utility is yet to be determined. The Kidney Solid Organ Response Test (kSORT) and whole-genome peripheral blood gene expression profiling assays are other blood biomarkers that use gene panels to identity rejection [47]. Larger validation studies are ongoing.

### 5.6. Imaging

Elastography is a non-invasive ultrasound technique that measures tissue fibrosis by analyzing the tissue’s response to the application of an external force [60]. Multiple studies have demonstrated that elastography can effectively evaluate the extent of early tubulointerstitial fibrosis in transplanted kidneys, showing strong correlation and reproducibility among observers [61,62]. However, further studies are needed to clarify its role in clinical practice.

## 6. Management

**Figure 3:**
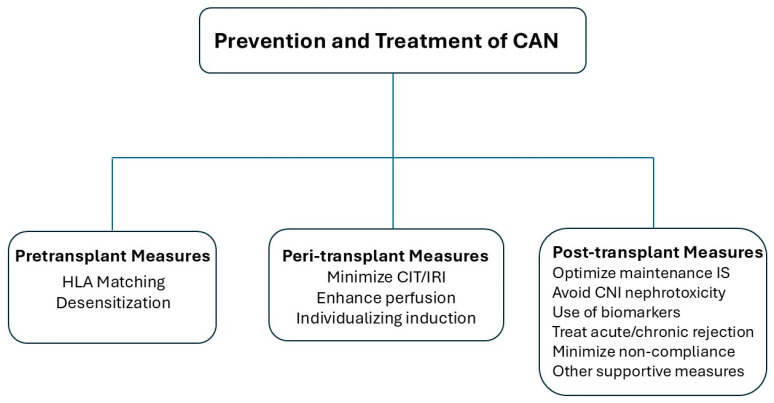
Prevention and treatment of CAN. HLA, human leukocyte antigen; CIT, cold ischemia time; IRI, ischemia reperfusion injury; IS, immunosuppression; CNI, calcineurin inhibitor.

Chronic allograft nephropathy represents the histologic manifestation of a series of time dependent factors. This suggests that several prevention and management strategies need to be employed, based in part on time from transplantation (Figure 3).

### 6.1. Pre-Transplant Measures

#### 6.1.1. HLA Matching

In the pre-transplant period, optimizing donor–recipient compatibility by HLA matching is one strategy to reduce allograft immunogenicity and prevent acute rejection. Improved HLA matching at the HLA-A,B and DR loci reduces the risk of acute rejection and improves graft survival [63,64]. There is also convincing evidence that mismatching at the DQ loci is associated with acute rejection and reduced graft survival, independent of standard HLA matching [65,66]. Recent findings have suggested that mismatches in HLA eplets, small amino acid sequences of the HLA molecule, are linked to acute rejection in kidney transplants, rather than broad HLA mismatches alone [67,68,69]. However, further research is needed to understand how to better define and interpret epitope compatibility before it can be applied in clinical practice.

#### 6.1.2. Desensitization in HLA-Incompatible Kidney Transplantation

Sensitization refers to the development of donor-specific antibodies (DSAs), commonly related to previous transplantation, pregnancy and blood transfusion. The presence of DSAs before transplantation can almost double the risk of antibody-mediated rejection and increase the likelihood of graft failure [70]. Transplant centers have employed a range of desensitization protocols based on their individual practices. Several studies have demonstrated good long-term allograft survival following HLA-incompatible living donor kidney transplantation [71,72]. In a multi-center study [73], 1025 kidney transplant recipients from HLA-incompatible live donors were compared with controls who either remained on the waiting list and received a transplant from a deceased donor or did not receive a transplant at all. The results showed that patients who received kidney transplants from HLA-incompatible live donors experienced a significant survival advantage, with a 5-year survival rate of 86%, compared to 59.2% in those who did not undergo transplantation and 74.4% in those who waited for a transplant from deceased donors (*p* < 0.001).

### 6.2. Peri-Transplant Measures

#### 6.2.1. Minimizing Cold Ischemia Time and Ischemia Reperfusion Injury (IRI)

Prior studies have consistently shown that prolonged cold ischemia time (CIT) is a risk factor for delayed graft function (DGF), acute renal transplant rejection (ARTR), and early allograft loss [74,75,76]. Following revascularization, tissue damage resulting from ischemia–reperfusion aggravates renal injury. Recent studies have shown an expected but undesirable outcome of increased CIT after the new kidney allocation system (KAS) implementation [77,78,79]. The impact of this on long-term graft outcomes is unknown but strategies to minimize cold ischemia may improve outcomes, especially in difficult-to-match organs with a higher kidney donor profile index (KDPI). The clinical benefits of hypothermic machine perfusion are well documented [80,81,82]. A novel method of organ preservation being increasingly used in kidney transplantation in the United States is normothermic regional perfusion (NRP). In this approach, donation after circulatory death (DCD) donors are placed on extracorporeal membranous oxygenation (ECMO) following the declaration of death to rapidly restore organ perfusion and reduce tissue ischemia prior to allograft recovery. Multiple studies, including a recent systematic review and meta-analysis, have shown that NRP as compared with standard in situ cold perfusion (ICP) in DCD organ kidneys is a safe and effective technique, potentially reducing DGF rates and improving early renal function [83,84,85,86,87]. In a large nationwide propensity score analysis conducted in Spain, Padilla et al. found that NRP was associated with better outcomes, including lower rates of DGF and 1-year graft loss compared to standard rapid recovery [88]. Similarly, in a retrospective analysis of NRP practices in the US, Merani et al. reported superior early kidney allograft function compared to standard recovery techniques [89]. Before this procurement technique can be used more widely, further research is needed to evaluate its long-term safety, efficacy, and costs.

#### 6.2.2. Individualizing Induction Immunosuppression

Induction agents administered around the time of transplant improve allograft survival by lowering the risk of acute rejection and potentially allowing for a reduction in maintenance immunosuppression, including CNIs. The categories of induction agents available include lymphocyte-depleting agents such as anti-thymocyte globulin (ATG) and Alemtuzumab (anti-CD52 monoclonal antibody) and non-depleting agents such as Basiliximab (IL-2R monoclonal antibody). The choice of induction agents depends on the immunologic risk of acute rejection. For patients with high immunologic risk, the use of ATG is beneficial to minimize the risk of acute rejection. For patients at lower immunologic risk, both ATG and Basiliximab are reasonable choices. Similar or lower rates of acute rejection have been observed in randomized trials comparing Alemtuzumab with either ATG or Basiliximab [90,91]. However, long-term outcomes, including CAN, may be worse in patients receiving Alemtuzumab [92,93]. An intense and sustained lymphopenia associated with Alemtuzumab may also contribute to its infrequent use.

### 6.3. Post-Transplant Measures

#### 6.3.1. Optimizing Maintenance Immunosuppression

Optimizing maintenance immunosuppression in the early post-transplant is crucial to prevent both acute and chronic rejection. For most patients, maintenance immunosuppressive therapy consists of a CNI, an antimetabolite, and prednisone. There is evidence to suggest that early steroid withdrawal (ESW) is associated with an increased risk of CAN [94,95]. However, in carefully selected patients with low immunologic risk, ESW can reduce long-term steroid-related side effects, with similar transplant outcomes [96,97,98,99].

#### 6.3.2. CNI Avoidance or Minimization Strategies

CNI nephrotoxicity can develop at any point after transplant and is not necessarily dose dependent. In the early post-transplant period, acute CNI nephrotoxicity can not only prolong DGF but also impede renal recovery stemming from other etiologies [100]. Its effects can be reversed with dose reduction or drug discontinuation. While overdiagnosis is a concern, chronic CNI nephrotoxicity is characterized by a progressive and irreversible decline in allograft function and can contribute to late graft loss. Among patients with established CAN receiving CNIs, there is a lack of evidence to suggest that CNI avoidance or minimization may be effective at preserving graft function. In addition, studies evaluating the use of CNI-free immunosuppression have shown that patients with higher Banff chronicity scores were more likely to be non-responders [101,102]. Where these strategies appear to be most effective is in the early post-transplant period, before the chronic structural damage associated with CNI nephrotoxicity has set in. A 2016 meta-analysis by Sawinski et al. [103] assessed various strategies to minimize CNI exposure. The analysis provided strong evidence that CNI minimization, involving low doses of cyclosporine or tacrolimus combined with mycophenolic acid or mTOR inhibitors, led to better renal function, a lower risk of BPAR, and reduced graft loss. This approach was found to be most effective when started early, within the first six months post-transplant.

Belatacept, a non-nephrotoxic and non-diabetogenic costimulation blocker, is an attractive approach for maintenance immunosuppression to avoid the toxicities associated with CNIs. In the BENEFIT trials [104,105,106], Vincenti et al. compared the use of de novo Belatacept- and Cyclosporine-based immunosuppression. Although the Belatacept group had a higher risk of acute rejection, primarily within the first-year post-transplant, the long-term benefits, including improved renal function and lower graft loss rates, persisted for up to seven years following transplant. Studies comparing the use of Belatacept with Tacrolimus are limited to only two randomized controlled trials (RCTs) to date. One such phase 3 trial evaluated the effectiveness of switching kidney transplant recipients from CNI-based to Belatacept-based maintenance immunosuppression at for least 6 months following transplant [107]. Results demonstrated similar graft survival, sustained improvement in renal function, a higher biopsy proven acute rejection (BPAR) rate, and a reduced incidence of de novo DSA at two years in the Belatacept conversion group. With tacrolimus being the preferred initial choice for immunosuppression after kidney transplant, further RCTs with Tacrolimus as the comparator are needed to determine whether the observed findings favoring Belatacept will improve clinically significant long-term outcomes.

#### 6.3.3. Treatment of Acute Rejection

Untreated acute rejection is a significant risk factor for the development of CAN. The likelihood of graft loss increases with severe rejection episodes, those occurring later post-transplant, and cases that do not respond effectively to treatment [108]. Treatment of acute cellular rejection differs across transplant centers. TCMR IA and IB are generally treated with high-dose steroids, while Thymoglobulin is usually reserved for TCMR II and III or for cases of TCMR IA/B that fail to respond adequately to initial therapy. Several studies have demonstrated an incomplete resolution of histologic changes after treatment, even in patients who achieve a complete recovery based on kidney function, highlighting the importance of follow up biopsies [109,110,111]. While there is a lack of consensus, follow-up biopsies are typically performed 2–4 weeks following treatment.

Treatment of antibody-mediated rejection (ABMR) consists of a combination of therapies, typically including high-dose steroids, plasmapheresis, and IVIG. The use of other therapies, including Rituximab and Bortezomib, varies between transplant centers. The timing of rejection also affects the approach to therapy. Early rejections tend to respond more to treatment [109,110,111] and many centers take an aggressive approach. ABMR that occurs late following transplant carries a worse prognosis, and more aggressive therapies have not been shown to halt progression of disease. Centers often take an individualized approach, weighing the risks of infection and malignancy with the benefits of therapy.

#### 6.3.4. Treatment of Chronic Rejection

Chronic active antibody-mediated rejection (caAMR) is one of the most common causes of late graft failure [112,113]. There are no standard treatments for managing caAMR and insufficient data exist to support the routine use of PLEX and IVIG, with or without Rituximab. Treatment approaches differ between transplant centers, but the expert consensus advocates for optimized immunosuppression and supportive care [114,115]. However, even this strategy is undergoing debate, based in part on the recent OuTSMART trial, a RCT that showed no improvement in graft failure with intervention to improve adherence and optimize immunosuppression in patients who developed de novo DSA [116]. The lack of new approved therapies is in part related to the challenges of recruitment; however, investigational agents are being studied. The IMAGINE study (NCT03744910) [117], a RCT evaluating the efficacy of the anti-IL6 antibody Clazakizumab in kidney transplant patients with caAMR, did show promise in phase 2 studies. However, the phase 3 trial was prematurely terminated after an interim analysis failed to show efficacy. Recent results from the phase 2 trial of Felzartamab, a CD-38 monoclonal antibody, showed promising therapeutic benefits in patients with late ABMR, warranting further investigation [118]. Trials evaluating late ABMR and caAMR are listed in Table 2.

Chronic active TCMR (CA TCMR) is also thought to be a leading cause of late graft loss [128] and is characterized by histologic findings of inflammatory infiltrates in atrophic areas (i-IFTA) in association with tubulitis. Prognosis largely depends on the severity and timing of diagnosis. There is some debate regarding the specificity of i-IFTA as a marker for previous rejection [129,130]. However, studies show that in a subset of patients, treating CA TCMR has the potential to improve graft function [128,131,132].

#### 6.3.5. Other Supportive Measures

Additional supportive measures to prevent the development of CAN include managing blood pressure, hyperlipidemia, and proteinuria. There is evidence to suggest that post-transplant hypertension is associated with poor long-term allograft survival [133,134]. There is a lack of high-quality evidence to recommend a BP target in kidney transplant recipients; however, a goal of <130/80 is generally advised [135]. While RCTs have not definitively demonstrated that ACE-I or ARB therapy improves patient or graft survival in kidney transplant recipients, their use may be beneficial for patients with proteinuria based on observational studies [136,137]. There are encouraging, albeit limited, data regarding the short-term outcomes of SGLT2I in kidney transplant recipients [138,139,140,141]. The ongoing RCTs INFINITI2019 (NCT04965935) [142] and CREST-KT (NCT04906213) [143] will hopefully shed further light on the long-term impacts of SGLT2i use in this population.

## 7. Conclusions

Chronic allograft nephropathy represents the histopathologic outcome of a series of time-dependent immune and non-immune insults together with the kidney healing response. These events are not independent of each other but are rather dynamic processes that often occur simultaneously. Management strategies should target the prevention and treatment of factors driving allograft injury. Personalized medicine, tailored to individual risk factors and patient characteristics, has the potential to optimize immunosuppression and enhance long-term graft survival. Early detection of graft dysfunction is critical when the graft function is still salvageable. Future directions include the implementation of AI-based diagnostic tools to better predict the long-term risk of allograft failure. Further research is needed to determine the clinical utility and cost effectiveness of novel strategies for the prevention and treatment of CAN.

## Figures and Tables

**Table 1:** Histologic criteria for chronic allograft nephropathy.

Grade	Histology	Interstitial Fibrosis (ci)	Tubular Atrophy (ct)
I	Mild	ci1: 6–25% of cortical area	ct1: Up to 25% of cortical tubules
II	Moderate	ci2: 26–50% of cortical area	ct2: 26–50% of cortical tubules
III	Severe	ci3: >50% of cortical area	ct3: >50% of cortical tubules

**Table 2:** Trials evaluating late ABMR and caAMR.

Study Design	Inclusion Criteria	Test Therapeutics	Patients	Follow Up	Major Results	Ref
RCT	DSA+ ABMR, ≥6 mo post-Tx, eGFR ≥ 20	Bortezomib	44	24 months	No difference	Eskandry et al. [119]
RCT	DSA+ ABMR, ≥6 mo post-Tx, eGFR ≥ 20	Felzartamab	22	52 weeks	Potential therapeutic benefit	Mayer et al. [118]
RCT	DSA+ caAMR, eGFR ≥ 20	IVIG + Rituximab	25	12 months	No difference (prematurely terminated)	Moreso et al. [120]
RCT	DSA+ caAMR, eGFR ≥ 20	Rituximab	47	3 years	No difference (prematurely terminated)	Shiu et al. [121]
Single group Assignment (Phase 2)	CAN or TG with c4d, >6 mo post-Tx, eGFR ≥ 20	Fostamatinib	10	52 weeks	Ongoing	Tam et al. [122]
RCT (Phase 2)	DSA+ ABMR, ≥12 mo post-Tx	Clazakizumab	20	52 weeks	Potential therapeutic benefit	Doberer et al. [123]
RCT (Phase 3)	DSA+ caAMR, ≥6 mo post-Tx	Clazakizumab	100	52 weeks	No difference (prematurely terminated)	Nickerson et al. [124]
RCT	DSA+ caAMR, ≥12 mo post-tx	Tocilizumab	50	24 months	Ongoing	Streichart et al. [125]
Single-arm Phase 1 trial	DSA+ ABMR, >6 mo post-tx, eGFR ≥ 20	Sutimlimab	10	50 days	No effect	Eskandry et al. [126]
RCT	DSA+, declining graft function, >6 mo post-tx	Eculizumab	16	52 weeks	Potential therapeutic benefit	Kulkarni et al. [127]

RCT, randomized control trial; DSA, donor-specific antibody; ABMR, antibody-mediated rejection; caAMR, chronic active antibody-mediated rejection; eGFR, estimated glomerular filtration rate.

## Data Availability

The data used in this article were sourced from the materials mentioned in the References section.
